# Influence of Electrical Transients and A/D Converter Dynamics on Thermal Resistance Measurements of Power MOSFETs

**DOI:** 10.3390/s25216691

**Published:** 2025-11-02

**Authors:** Krzysztof Górecki, Krzysztof Posobkiewicz

**Affiliations:** Department of Power Electronics, Gdynia Maritime University, Morska 81-87, 81-225 Gdynia, Poland; k.posobkiewicz@we.umg.edu.pl

**Keywords:** A/D converters, electrical transients, indirect electrical method, measurements, power MOSFETs, thermal resistance, thermal time constants

## Abstract

**Highlights:**

**What are the main findings?**

- An analysis of datasheets of power MOSFETs indicates that the accuracy of thermal resistance measurements depends on the shortest thermal time constants of the tested transistors.
- Measurements performed using the system built by the authors indicate that electrical transients and oscillations are observed for up to several milliseconds, which may significantly affect measurement accuracy.
- The measurement error of thermal resistance is generally higher for power MOSFETs made of silicon carbide and gallium nitride than for silicon transistors.

**What is the implication of the main finding?**

- The measurement of the thermal sensitive electrical parameter (TSEP) during thermal resistance measurements of power MOSFETs should start only after electrical transients and oscillations have faded out. In the investigated system, this occurs up to 2.5 ms after the switchover from transistor heating to TSEP recording.
- The measurement error of thermal resistance for power MOSFETs made of silicon carbide is higher than for devices made of silicon or gallium nitride.

**Abstract:**

When designing power electronic systems, it is crucial to correctly estimate the junction temperature of semiconductor devices, particularly power MOSFETs, under actual operating conditions. Thermal resistance is a parameter that characterizes the ability of these devices to dissipate internally generated heat under steady-state conditions. Determining the value of this parameter under specific cooling conditions requires dedicated measurements. This paper considers the widely used indirect electrical method of measuring thermal resistance. The influence of the dynamic properties of the measurement system, including the A/D converter, on the measurement error of the thermal resistance of power MOSFETs was analyzed. Using the constructed measurement system, it was demonstrated that, depending on the semiconductor material of the tested transistors, different error values were obtained, even with the same system configuration. The largest errors were observed for transistors made of silicon carbide. It was further shown that, with the applied A/D converter module, the measurement error can be limited to a few percent if recording of the thermal sensitive electrical parameter (TSEP) begins soon enough after the transients caused by the switchover from heating to TSEP measurement have fully decayed.

## 1. Introduction

MOSFETs are currently the most widely used semiconductor devices in power electronic systems operating at voltages of up to several hundred volts [1,2]. These devices are fabricated from different semiconductor materials, such as silicon (Si), silicon carbide (SiC), and gallium nitride (GaN) [3,4]. During operation, their junction temperature (*T_j_*) is higher than the ambient temperature (*T_a_*) due to self-heating [5]. The difference between *T_j_* and *T_a_* under steady-state conditions is determined by the thermal resistance (*R_th_*) [6,7,8] of the transistor and the power losses (*P*) within the device.

Thermal resistance characterizes the ability of a semiconductor device to dissipate internally generated heat to its surroundings [9]. Its value depends on the properties of the materials used in the device structure, the mounting method, and the cooling system, which may be passive or active [10,11]. Power MOSFET manufacturers provide thermal resistance values in datasheets; however, these values are limited to boundary conditions, i.e., ideal case cooling (*R_thj-c_*) or operation at natural convection of the device case (*R_thj-a_*) [12]. Under real operating conditions, the actual thermal resistance lies between these extremes. Determining the *R_th_* value under specific conditions (mounting method, type of cooling system, etc.) is therefore necessary, for example, to optimize the cost and design of the cooling solution [13].

Typically, Rth is measured using the indirect electrical method [12,14]. This method relies on the dependence of a selected electrical parameter—the so-called thermal sensitive electrical parameter (TSEP)—on the junction temperature of the device [15,16]. The procedure involves heating the device under test until thermal steady state is reached, and then measuring the TSEP during the cooling process. Due to the step-like change in the operating point of the transistor, both electrical and thermal transients occur simultaneously in the system.

For power MOSFETs, the following parameters are used as TSEP [7]: the gate-source voltage V_GS_ for the transistor operating in the saturation range at a fixed drain current, the voltage V_F_ across the forward-biased diode at a fixed forward current, or the on-channel resistance R_DSON_. Each of the mentioned parameters has a different temperature dependence and sensitivity [17]. In particular, the use of R_DSON_ as TSEP for transistors made of SiC or GaN is a significant problem due to the very low value of this parameter—comparable to the resistance of the leads and connections [18]. Therefore, further considerations are limited to the V_GS_ voltage acting as TSEP.

Measuring the TSEP after heating requires a step change in the current flowing through the transistor [19,20]. Because this change induces both electrical and thermal transients, it is necessary to wait a certain period before measurement. This delay should be long enough to allow electrical transients to vanish, but short enough to minimize the reduction in junction temperature.

The problem of applying indirect electrical methods is widely described in the literature, e.g., in [21,22,23,24,25,26,27,28,29,30,31,32,33,34,35,36,37,38]. Paper [21] concerns high-resolution temperature mapping in a p-GaN HEMT die while dissipating high-density power in this die. Measurements were performed using thermoreflectance microscopy. Paper [22] concerns submicron temperature mapping of AlGaN/GaN HEMTs under low and high current conditions. The usefulness of Raman thermography for comparing the accuracy of optical and electrical methods was demonstrated. Paper [23] presents the results of dynamic thermoreflectance studies for the gate temperature in GaN HEMTs operating under switching conditions.

In turn, in [24], an indirect electrical method for determining the junction temperature and *R_th_* in GaN HEMTs was presented. It was indicated that the proposed measurement method is useful when optical methods are difficult to use, e.g., for transistor dies mounted in packages. In [25], the problem of determining the thermal resistance of GaN HEMTs using an indirect electrical method is considered, which utilizes parameters such as the *V_GS_* voltage and the gate resistance R_G_. The cited paper also discusses the advantages and disadvantages of using other TSEPs and calibration procedures. Paper [26] describes a technique for measuring the transient thermal impedance *Z_th_(t)* of a GaN HEMT based on frequency separation of the thermal response by measuring the gate resistance. Paper [27] analyzes the problem of monitoring the transient thermal response of AlGaN/GaN HEMTs. The research used the transient thermoreflectance imaging method with a switched power supply.

In [28], the problem of measuring *Z_th_(t)* of GaN HEMTs was described. The transient thermal thermoreflectance method was used in the measurements. The studies were carried out over a wide range of ambient temperatures. The measurement results were compared with the results of FEM modeling and compact modeling. In [29], the analysis of thermal parameters (*R_th_*, *Z_th_(t)*) of packaged GaN transistors is considered. The considerations are carried out for different cooling systems and also include studies of the quality of the semiconductor die assembly process in the packages.

Paper [30] presents a method for measuring *Z_th_(t)* of GaN HEMTs with a p-type gate. In the presented method, the *V_GS_* voltage is used as the TSEP. Paper [31] describes the methodology for measuring *Z_th_(t)* and determining the thermal time constants of GaN HEMTs using Raman microscopy. Paper [32] considers the problem of determining Rth of a GaN HEMT using de-trapping characteristics. Drain-current recovery times are used for this purpose. Paper [33] presents a fast method for measuring *R_th_* of a GaN HEMT using TSEP. The studies were carried out for a high V_DS_ value. Paper [34] indicates such thermally sensitive parameters of GaN HEMT transistors as threshold voltage, substrate diode forward voltage, and saturation voltage. The measurement sensitivity using each of the considered parameters was analyzed.

In [35], a method for measuring the thermal resistance of GaN HEMTs using the modulation method was described. The *V_GS_* voltage and the *R_DSON_* resistance were used as the TSEP. In [36], a method for monitoring the transistor junction temperature was described using the maximum value of the *di/dt* derivative as the TSEP. Paper [37] describes a method for determining the thermal resistance of GaN HEMTs based on measured dynamic characteristics of the transistor’s output conductance. Paper [38] proposes a high-accuracy thermal resistance measurement method for GaN HEMTs based on a heating power modulation strategy, called the harmonic pulse width sub-threshold (HPWS) method. The sensitive linear correlation between the sub-threshold swing of GaN HEMTs and temperature is utilized, and the turn-off transients of the heating power signal are sampled to extract the thermal resistance through frequency domain scanning of the heating signal modulation.

As it is visible from the presented literature review, very often *V_GS_* voltage is used as the TSEP. Classical studies [39,40] indicate that, for silicon transistors, the TSEP can reasonably be measured at two points in time (100 μs and 200 μs), with extrapolation of the TSEP(t) curve back to t = 0 using a square-root approximation. The classical square root extrapolation of the measured values of TSEP cannot be usable if the waveform TSEP(t) includes oscillations and the value of TSEP is measured in two moments only. Analysis of datasheets of power MOSFETs made of wide bandgap materials (GaN, SiC) [13,14,15] shows that thermal processes in these devices occur faster than in silicon transistors. Therefore, the dynamic properties of the measurement system are expected to have a significant influence on the obtained *R_th_* values [20,41].

The aim of this paper is to investigate the influence of the electrical time constants of the transistor biasing circuit and the dynamic properties of the A/D converter module in the measurement system on the accuracy of thermal resistance measurements of power MOSFETs fabricated from different semiconductor materials. The systematic studies demonstrating that the thermal resistance measurement error depends significantly on all considered factors were conducted. Furthermore, it was theoretically and experimentally investigated, compared, and evaluated the *R_th_* measurement errors for transistors made not only of silicon but also of modern wide-bandgap materials.

Section 2 describes the measurement method and system. Section 3 presents the relationships between the measurement system parameters and the thermal properties of the tested transistors, and their influence on the *R_th_* measurement error. Section 4 provides the results of experimental studies on selected transistors.

## 2. Measurement Method and System

The thermal resistance of power MOSFETs is typically measured using the indirect electrical method [39,42]. The role of the thermal-sensitive electrical parameter (TSEP) can be played, for example, by the gate-to-source voltage *v_GS_* at a fixed drain current in the saturation region, or by the forward voltage v_F_ of a forward-biased freewheeling diode at a fixed current value [17,42]. For both TSEPs, the nonlinear thermometric characteristics TSEP (*T_j_*) are observed. The slope of these characteristics, and thus the measurement sensitivity, is higher when *v_GS_* is used as the TSEP [17].

**Figure 1:**
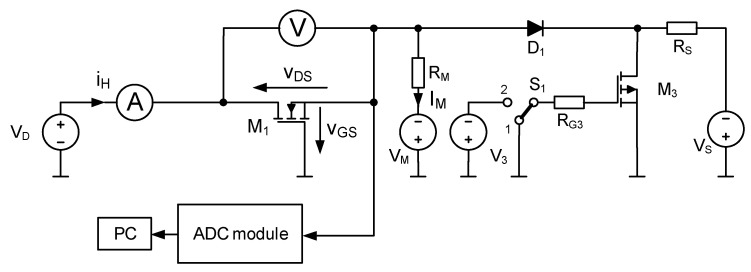
Diagram of the system for measuring *R_th_* of power MOSFETs.

The measurement system used in this study is shown in Figure 1. It enables *R_th_* measurements with *v_GS_* as the TSEP. In the figure, transistor M_1_ is the device under test (DUT).

The system consists of three main functional blocks: a DUT biasing circuit, an ADC module, and a PC. The biasing circuit sets the operating point of the tested MOSFET during the measurement procedure.

In this system, the USB-2404-60 ADC module manufactured by Measurement Computing (Norton, MA, USA) is used to acquire *v_GS_* [43]. This module has four independent measurement channels, each equipped with a 24-bit delta-sigma ADC, and supports sampling rates from 1.613 kS/s to 50 kS/s. The input voltage range is ±60 V. The module is controlled using a PC with the proprietary MCScan software [17,43]. An ammeter and voltmeter included in the system play a supplementary role, measuring the power losses in the DUT during the heating phase.

The Rth measurement procedure consists of several steps:

1. Measuring the thermometric characteristic *V_GS_(T_j_)* of the DUT placed in a thermostatic chamber, biased at the operating point Q_M_ = (*V_DSM_*, *I_M_*), with switch S_1_ in position 2.
2. Performing nonlinear regression to calculate the coefficients *a*, *b*, and *c* of a quadratic function approximating the dependence *T_j_*(*V_GS_*).
3. Heating the DUT until thermal steady state is reached (transistor biased at Q_H_ = (*V_DSH_*, *I_H_*), with power losses *P* = *V_DSH_·I_H_*, switch S_1_ in position 1).
4. Switchover of the biasing circuit from heating to TSEP recording by changing the position of switch S_1_ from 1 to 2. This changes the operating point of the transistor from Q_H_ to Q_M_ = (*V_DSM_*, *I_DM_*). At this step, the value *V_GSH_* corresponding to the junction temperature is recorded immediately after the switchover.
5. Calculating *R_th_* from the measurement results using the following formula [17]:
  (1) $$R_{th}=\frac{\left(a\cdot V_{GSH}^{2}+b\cdot V_{GSH}+c\right)-T_{a}}{V_{DSH}\cdot I_{H}},$$
  where *V_GSH_* is the gate-to-source voltage measured as soon as possible after the switchover of the measurement system.

During the transition from transistor heating to TSEP recording, both electrical and thermal transients occur due to the change in the operating point of the DUT and the presence of parasitic inductances and capacitances in the system. In practice, decaying oscillations of *v_GS_* are observed after the switchover. The *v_GS_* value used for the *R_th_* calculation (*V_GSH_*) can be reliably determined only once these oscillations have subsided.

## 3. Estimation of the Measurement Error

Considerations on the error in measuring the thermal resistance of power semiconductor devices using the indirect electrical method are presented, among others, in [17,44]. Using the total differential method, it was shown that the relative error in measuring the thermal resistance *R_th_* is the sum of the relative errors in measuring the temperature rise (*T_j_ − T_a_*) and the dissipated power.

The error in determining the temperature rise depends on the accuracy of approximating the *T_j_*(*v_GS_*) characteristic of the tested transistor and on the accuracy of the *V_GSH_* measurement. The latter is related not only to the resolution of the ADC module but also to the value of the voltage *V_GSH_* used for the *R_th_* calculation. Since this value can be obtained only after the decaying oscillations have ceased, the measurement is delayed by a time *t_d_*. While the error component resulting from the resolution of the ADC is small and easy to quantify, the error component caused by the delay depends on how much *T_j_* decreases before a reliable TSEP measurement can be performed. This decrease is determined by the steady-state value of *T_j_* after heating the transistor up and by the thermal time constants of the transistor and its cooling system. These thermal time constants characterize the dynamics of the heating and cooling process of the tested transistor. In particular, after a sudden shutdown of the power dissipated in the transistor, its junction temperature begins to decrease. The rate of temperature decline depends on the values of the thermal time constants. The smaller these values, the greater the temperature drop after the same time t_d_, and consequently, the greater the measurement error.

As shown in [45,46], thermal time constants span a very wide range (from microseconds to thousands of seconds) and are associated with individual elements of the heat flow path (semiconductor die, package, PCB, heat sink, etc.). Only the shortest thermal time constants—those related to the semiconductor structure—have a real influence on the Rth measurement error. To estimate this error, the classical approximation of the transient thermal impedance *Z_th_(t)* [46,47] was used. The time response of *Z_th_(t)* corresponds to the time response of the junction temperature for a step power dissipation. It can be expressed as follows:

(2) $$Z_{th}\left(t\right)=R_{th}\cdot \left(1-\sum_{i=1}^{N}a_{i}\cdot \exp\left(-\frac{t}{\tau_{thi}}\right)\right),$$

where *a_i_* is the weighting factor associated with the thermal time constant *τ_thi_*, and *N* is the number of thermal time constants.

The absolute error in determining the temperature rise *T_j_-T_a_* caused by a delay t_d_ in measuring the *V_GSH_* can be estimated as follows:

(3) $$\Delta T_{j1}=V_{DSH}\cdot I_{H}\cdot R_{th}\cdot \sum_{i=1}^{N}a_{i}\cdot \left(1-\exp\left(-\frac{t_{d}}{\tau_{thi}}\right)\right)$$

Formula (3) results directly from the observation that during a step change in the power dissipated in the transistor, the increase in its internal temperature is the product of the dissipated power value and the waveform *Z_th_(t)*. The power dissipated in the transistor during its heating is the product of the current I_H_ and the voltage *V_DSH_*, and the waveform *Z_th_(t)* is described by formula (2).

Equation (3) shows that as the delay t_d_ increases, the error in determining the temperature rise also increases. The error becomes larger for higher values of *a_i_* and for smaller values of *τ_thi_*. A large *a_i_* value is typical for transistors cooled with efficient systems, e.g., a heat sink with a fan or a liquid cooling system. The value of *τ_thi_* depends on the material and dimensions of the semiconductor structure and on the packaging technology.

## 4. Investigation Results

To estimate the influence of the delay time t_d_ on the *R_th_* measurement error for power MOSFETs made of different materials, datasheet waveforms of *Z_th_(t)* under ideal cooling conditions were analyzed. Three transistors in TO-247 packages were tested: silicon (Si), silicon carbide (SiC), and gallium nitride (GaN). The devices were IXFH15N100Q3 (Hi-perFET Q3-class produced by IXYS, Milpitas, California, CA, USA), C3M0280090D (C3M MOSFET Technology produced by Wolfspeed, Research Triangle Park, North Carolina, NC, USA), and TP65H050G4WS (GaN cascode produced by Transphorm, Goleta, California, CA, USA).

### 4.1. Properties of Tested Devices

The properties of the selected transistors are presented in [48,49,50]. Their maximum drain–source voltage ratings range from 650 V to 1000 V, the maximum drain current from 11.5 A to 34 A, and the on-state resistance *R_ON_* from 0.06 Ω to 1.05 Ω. The junction-to-ambient thermal resistance *R_thj-a_* specified by all manufacturers, is 40 K/W. The junction-to-case *R_thj-c_* thermal resistance of the selected transistors differs significantly. It is 0.18 K/W for the silicon transistor, 1.05 K/W for the GaN transistor, and 2.3 K/W for the SiC transistor.

**Figure 2:**
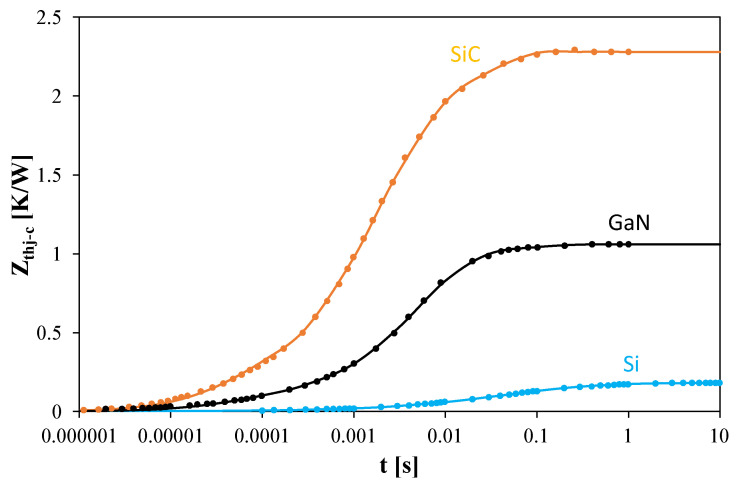
Datasheet *Z_th_(t)* waveforms of the tested power MOSFETs under ideal cooling, and their approximations.

Figure 2 shows the *Z_thj-c_(t)* waveforms from datasheets (points) together with the approximations obtained using formula (2) (lines). The silicon transistor is shown in blue, the GaN transistor in black, and the SiC transistor in orange. The parameters describing the *Z_th_(t)* waveforms were determined using the method in [47], and are summarized in Table 1.

The results in Figure 2 indicate that *Z_th_(t)* of the silicon transistor remains negligible for t < 2 ms, while for the GaN transistor, it is negligible for t < 20 μs, and for the SiC transistor for t < 3 μs. Table 1 shows that the shortest thermal time constants range from 40 μs to 200 μs. The number of thermal time constants describing the semiconductor structures and packages of the tested transistors ranges from 4 to 5, with the longest exceeding 600 ms.

### 4.2. Influence of Recording Delay on Junction Temperature Measurement Error

To evaluate the effect of t_d_ on the error in determining the junction temperature rise, error values *ΔT_j1_* were calculated for selected power dissipation levels in each transistor using formula (3). Ideal package cooling and selected values of t_d_ were considered. The results are presented in Table 2.

The absolute error in determining the junction temperature rise increases with both *t_d_* and *P*, but the magnitude of this increase differs among the tested transistors. Under the considered conditions, the highest errors occur for the SiC device. For *P* = 50 W and *t_d_* = 3 ms, the error exceeds 75 K, corresponding to a relative error greater than 66%. For the same *t_d_*, the error is about 20% for the silicon transistor and nearly 50% for the GaN transistor. At *t_d_* = 0.1 ms, the error ranges from 4% for Si to 14% for SiC.

Table 3 and Table 4 contain the values of the *ΔT_j1_* error calculated for selected values of the delay *t_d_* and dissipated power for the tested transistors operating in actual conditions. Table 3 corresponds to transistors operating at natural convection, whereas Table 4—to transistors situated on the heat sink characterized by *R_thc-a_* = 7 K/W.

These results highlight the importance of minimizing t_d_ in *R_th_* measurements. They also show that modern wide-bandgap MOSFETs (SiC, GaN), characterized by very short thermal time constants, are more susceptible to significant errors. By analyzing the data contained in Table 2, Table 3 and Table 4, it can be easily observed that the influence of time t_d_ on the error of the junction temperature measurement *ΔT_j1_* is stronger the higher the value of the power dissipated in the tested transistor and the more effective the cooling system is used.

The above discussion concerns the worst-case scenario of ideal case cooling, in which the devices cool down rapidly after switchover. In practical applications, cooling is less efficient, resulting in higher *R_th_* values, smaller weighting factors for the shortest time constants, and additional longer time constants associated with other elements of the heat flow path [17,46]. These longer time constants (seconds to thousands of seconds) have a negligible influence on *ΔT_j1_*.

Table 5 shows the relative error *ΔT_j1_/(T_j_ − T_a_)* for each transistor operating under two cooling conditions: without a cooling system (*R_th_* = 40 K/W) and with a medium-sized heat sink (*R_th_* = 7 K/W + *R_thj-c_*).

The results show that the relative error increases with both t_d_ and the efficiency of the cooling system (lower *R_th_*). Among the tested devices, the SiC transistor exhibits the largest error. With a heat sink and *t_d_* > 1 ms, the error exceeds several percent, and for ideal cooling of the transistor case, this error could exceed even 40%. For the silicon transistor, the error remains below 0.5% under all actual cooling conditions, which is acceptable. To achieve below 1% error for SiC and GaN devices operating at actual cooling conditions, *t_d_* must be shorter than 0.1 ms.

### 4.3. Influence of ADC Properties on TSEP Recording Delay

This section presents measurement results of the thermally sensitive parameter for the considered transistors under different cooling conditions and ADC configurations (sampling period T_S_ and recording time t_R_). In all graphs, t = 0 corresponds to the switchover from heating to TSEP recording.

**Figure 3:**
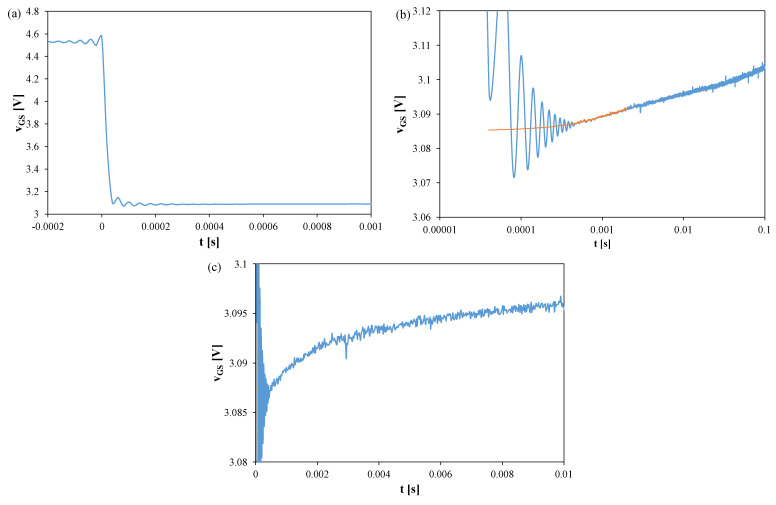
Measured (blue) and approximated (orange) waveforms of the *v_GS_* voltage of the silicon MOSFETs without a heat sink; T_S_ = 20 μs, t_R_ = 100 ms: (**a**) *v_GS_* changes during the switchover from heating to TSEP recording, (**b**) settling of the *v_GS_*, logarithmic time scale, (**c**) settling of the *v_GS_*, linear time scale.

**Figure 4:**
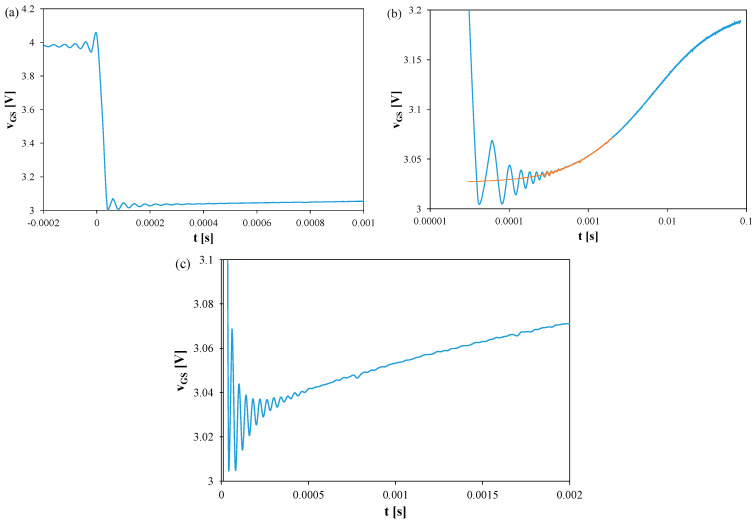
Measured and approximated waveforms of the *v_GS_* voltage of the GaN MOSFETs operating without any heat sink; T_S_ = 20 μs, t_R_ = 100 ms: (**a**) *v_GS_* changes during the switchover from heating to TSEP recording, (**b**) settling of the *v_GS_*, logarithmic time scale, (**c**) settling of the *v_GS_*, linear time scale.

**Figure 5:**
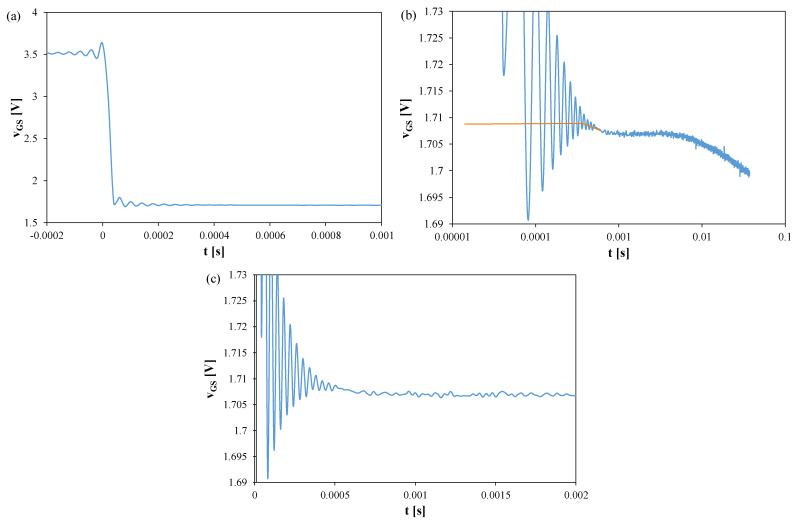
Measured and approximated waveforms of the *v_GS_* voltage of the SiC MOSFETs without a heat sink; T_S_ = 20 μs, t_R_ = 100 ms: (**a**) *v_GS_* changes during the switchover from heating to TSEP recording, (**b**) settling of the *v_GS_*, logarithmic time scale, (**c**) settling of the *v_GS_*, linear time scale.

Figure 3, Figure 4 and Figure 5 show *v_GS_* waveforms for Si (Figure 3), GaN (Figure 4), and SiC (Figure 5) transistors recorded without a heat sink at *P* = 4 W, with T_S_ = 20 μs and t_R_ = 100 ms. Additionally, the approximations of these waveforms, *v_GS_(t)*, are shown in Figure 3b, Figure 4b, and Figure 5b, respectively, and marked with orange color.

In each case, fading oscillations of *v_GS_* are observed. Their amplitudes correspond to junction temperature fluctuations of 15–25 K, disappearing after 0.3–0.5 ms. For the SiC transistor, a negative slope of *v_GS_* is also visible due to the electron trapping effect [51,52]. The orange line indicates a function approximating the measured *v_GS_(t)* waveform using a quadratic function. This line can be used to extrapolate the *v_GS_(t)* dependence and determine the *v_GS_* value corresponding to time t = 0.

Analyzing the waveforms shown in Figure 5, it can be seen that the measured *v_GS_* voltage values for this transistor are almost half that of the other transistors. It is also visible that after switching the measurement system to TSEP recording mode, the measured *v_GS_* voltage decreases instead of increasing. This is caused by the trapping effect described, among others, in [51,52]. It is worth adding that in the case of this transistor, it is worth taking into account that the *V_GSH_* value used to calculate *R_th_* should be read only when the *v_GS_(t)* relationship becomes an increasing function.

**Figure 6:**
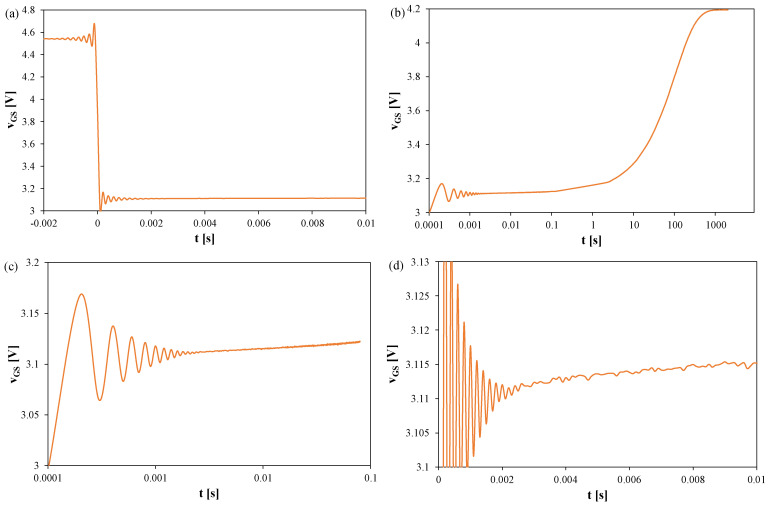
Measured waveforms of the *v_GS_* voltage of the silicon MOSFETs without any heat sink; T_S_ = 100 μs, t_R_ = 2000 s: (**a**) *v_GS_* changes during the switchover from heating to TSEP recording, (**b**) the entire recording range, (**c**) settling of the *v_GS_*, logarithmic time scale, (**d**) settling of the *v_GS_*, linear time scale.

**Figure 7:**
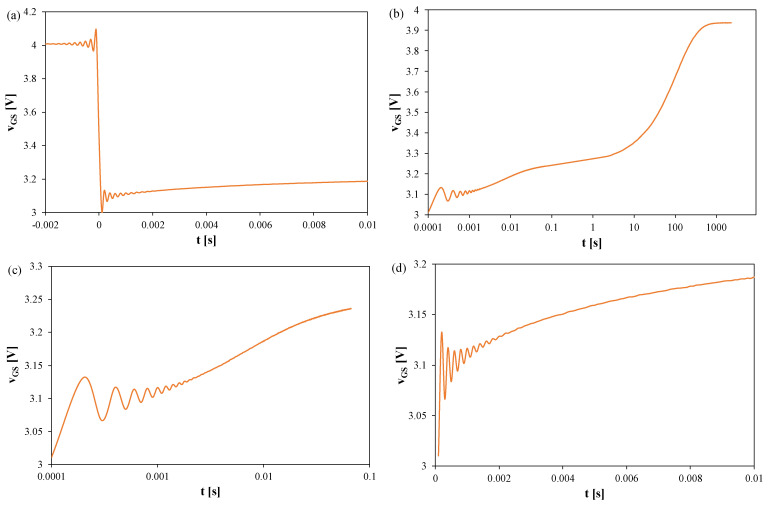
Measured waveforms of the *v_GS_* voltage of the GaN MOSFETs operating without a heat sink; T_S_ = 100 μs, t_R_ = 2000 s: (**a**) *v_GS_* changes during the switchover from heating to TSEP recording, (**b**) the entire recording range, (**c**) settling of the *v_GS_*, logarithmic time scale, (**d**) settling of the *v_GS_*, linear time scale.

**Figure 8:**
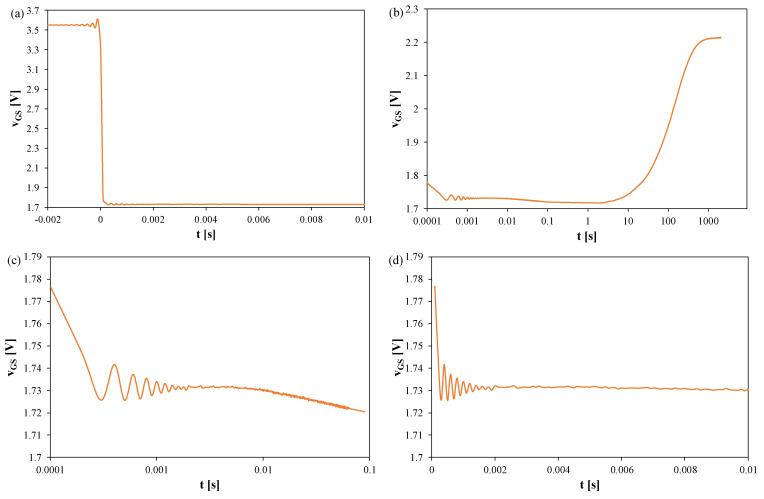
Measured waveforms of the voltage *v_GS_* of the SiC MOSFETs operating without any heat sink; T_S_ = 100 μs, t_R_ = 2000 s: (**a**) a fragment of the waveform immediately after switching the transistor current, (**b**) the entire recording range, (**c**) magnification on a logarithmic time scale, (**d**) magnification on a linear time scale.

Figure 6, Figure 7 and Figure 8 present the *v_GS_* waveforms for the same devices without a heat sink, but with T_S_ = 100 μs and t_R_ = 2000 s. The thermal steady state occurs after about 800 s in all cases. Oscillation amplitudes range from 50 mV (SiC) to 170 mV (Si), corresponding to 25–60 K, and decay only after about 2–2.5 ms. For the SiC transistor, the increase in *v_GS_* becomes visible only for t > 1 s, implying *t_d_* ≈ 1 s.

In Figure 6, it can be observed that the thermal steady state occurs after about 800 s from the moment the measuring system is switched to the TSEP recording mode. The peak-to-peak value of the oscillations reaches 170 mV, which corresponds to about 60 K. These oscillations disappear only after 2.5 ms.

In Figure 7, it can be observed that the thermal steady state occurs after about 800 s from the moment the measuring system is switched to the TSEP recording mode. The peak-to-peak value of the oscillations reaches 120 mV, which corresponds to about 35 K. These oscillations disappear only after 2 ms.

In Figure 8, it can be observed that the thermal steady state occurs after about 800 s from the moment of switching the measuring system to the TSEP recording mode. The peak-to-peak value of the oscillations reaches 50 mV, which corresponds to about 25 K. These oscillations disappear only after 2 ms. It is worth noting that in the case of this transistor, the increase in the *v_GS_* voltage value is visible only in the time interval t > 1s. According to the considerations presented above, for this transistor, the time *t_d_* = 1 s.

**Figure 9:**
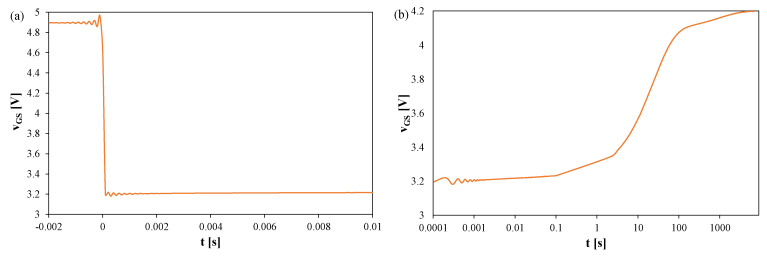

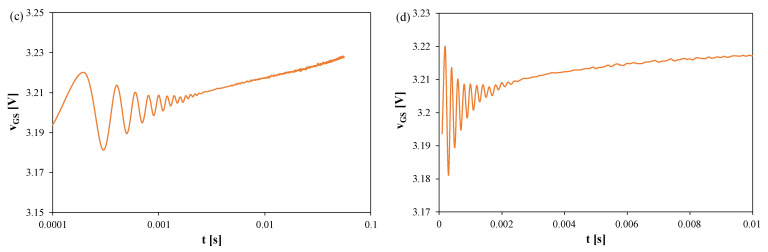
Measured waveforms of the voltage *v_GS_* of the silicon MOSFETs on a heat sink; T_S_ = 100 μs, t_R_ = 2000 s: (**a**) a fragment of the waveform immediately after switching the transistor current, (**b**) the entire recording range, (**c**) magnification on a logarithmic time scale, (**d**) magnification on a linear time scale.

**Figure 10:**
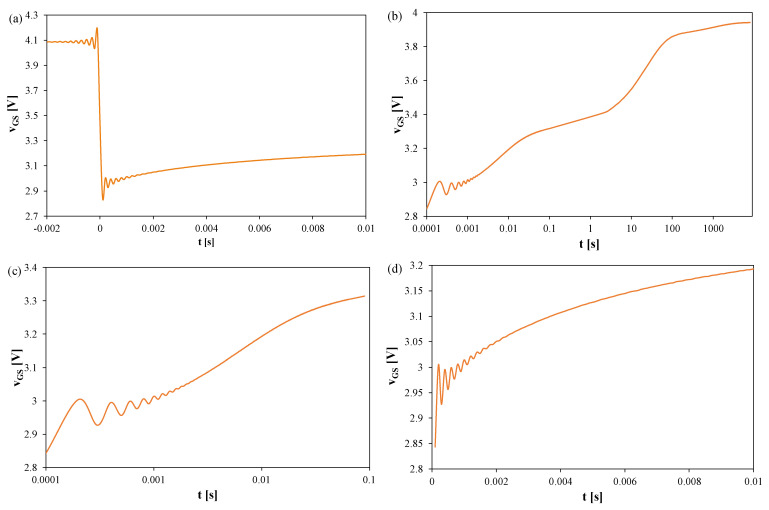
Measured waveforms of the voltage *v_GS_* of the GaN cascode on a heat sink; T_S_ = 100 μs, t_R_ = 2000 s: (**a**) fragment of the waveform immediately after switching the I_H_ current, (**b**) the entire recording range, (**c**) magnification on a logarithmic time scale, (**d**) magnification on a linear time scale.

**Figure 11:**
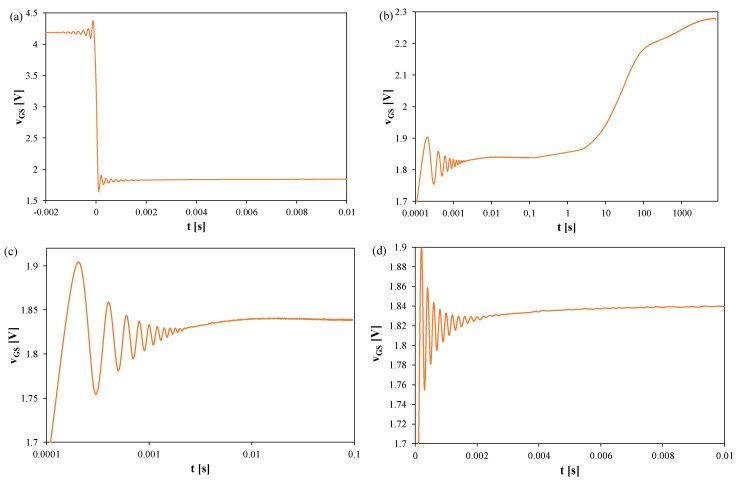
Measured waveforms of the voltage *v_GS_* of the SiC MOSFETs on a heat sink; T_S_ = 100 μs, t_R_ = 2000 s: (**a**) a fragment of the waveform immediately after switching the transistor current, (**b**) the entire recording range, (**c**) magnification on a logarithmic time scale, (**d**) magnification on a linear time scale.

Figure 9, Figure 10 and Figure 11 show the waveforms of the *v_GS_* voltage of Si (Figure 9), GaN (Figure 10), and SiC (Figure 11) transistors recorded using the measurement setup. The presented results refer to transistors operating on a heat sink, in which the power dissipation was 9 W. The sampling period T_S_ = 100 μs, and the recording time was 2000 s.

In Figure 9, it can be observed that the thermal steady state is reached after approximately 7000 s from the moment the measuring setup is switched to the TSEP recording mode. The peak-to-peak value of the oscillations reaches 40 mV, which corresponds to approximately 12 K. These oscillations disappear only after 2 ms.

In Figure 10, it can be observed that the thermal steady state occurs after about 7000 s from the moment the measuring system is switched to the TSEP recording mode. The peak-to-peak value of the oscillations reaches 160 mV, which corresponds to about 45 K. These oscillations disappear only after 1.8 ms.

In Figure 11, it can be observed that the thermal steady state is reached approximately 8000 s after switching the measuring setup to the TSEP recording mode. The peak-to-peak value of the oscillations reaches 200 mV, which corresponds to about 100 K. These oscillations disappear only after 2 ms. The section of the *v_GS_(t)* waveform with a negative slope, which occurs when this transistor is operated without a heat sink, is not visible.

The ADC module uses both analog and digital filtering. The effective bandwidth depends on the selected sampling period. Oscillations are visible in both cases (T_S_ = 20 μs and T_S_ = 100 μs), but they persist longer with the larger T_S_. Specifically, oscillations decay after ~500 μs for T_S_ = 20 μs and after ~2 ms for T_S_ = 100 μs, indicating that the dynamic properties of the ADC affect the apparent settling time.

### 4.4. Influence of Properties of ADC Module on a Measurement Error

Combining the previous results, it can be concluded that the sampling period of the ADC strongly influences the time required for oscillations of *v_GS_* to disappear. According to formula (3), increasing t_d_ increases the measurement error of the junction temperature rise, and thus the relative error in *R_th_*.

In the Figures shown in the previous sections, it is visible that the oscillations on the waveforms *V_GS_(t)* can be observed for a time longer than 400 μs. These oscillations are the result of both the dynamic properties of the used circuit polarizing the tested transistor and the properties of the used ADC module. If the value of *V_GSH_* voltage is measured at one of the time moments in which the high oscillations are observed, a big measurement error can be obtained. Additionally, if any extrapolation of *V_GSH_* voltage corresponding to time t = 0 will be performed using measured values of *V_GS_* voltage in the time interval in which the oscillations are observed, a big measurement error can also be obtained. Therefore, the values of *V_GSH_* should be measured when the mentioned oscillations are not observed.

Assuming that other error components (ambient temperature accuracy, TSEP calibration, and power loss determination) are negligible compared to this effect, the relative Rth error δ_Rth_ was calculated for the three devices under different cooling conditions and ADC sampling periods. The results are summarized in Table 6.

The results show that extending the sampling period increases the measurement error. Improved cooling conditions also increase the error because they accelerate the temperature decrease during the oscillation decay. The largest errors occur for SiC transistors situated on the heat sink. This error exceeds even 19% for T_S_ = 100 μs. In contrast, the error obtained for a transistor made of Si does not exceed 3.2%.

It should be noted that these error values were calculated assuming a sufficiently large *T_j_* − *T_a_* difference to neglect errors due to ambient temperature measurement, thermometric characteristic approximation, and power loss determination. As shown in [23,25], these error components together do not exceed 4% when *T_j_* − *T_a_* > 80 K.

## 5. Conclusions

This paper presented research results illustrating the influence of the dynamic properties of the ADC module and the transistor biasing circuit on the measurement error of the thermal resistance of power MOSFETs fabricated from different semiconductor materials. Particular attention was given to the oscillations occurring in the measurement system immediately after the switchover from heating to TSEP recording. The TSEP value can be measured reliably only after these oscillations decay, during which time the junction temperature of the device decreases.

The problems specific to thermal resistance measurements of the mentioned devices, compared to classic silicon components, were presented. It is also important to demonstrate that in transistors made of silicon carbide or gallium nitride, thermal phenomena in these components occur more rapidly than in silicon transistors. Therefore, to obtain sufficiently accurate results, main attention should be paid to the measurement equipment used, especially the ADC module, and the selection of the sampling period.

The measurements demonstrated that the time required for oscillations to vanish increases with the sampling period of the ADC module. Another important factor affecting measurement accuracy is the cooling efficiency of the tested device. High-efficiency cooling accelerates the decrease in junction temperature during oscillation decay, which increases the measurement error. The cooling rate depends on the cooling method, the semiconductor material, and the packaging technology. The highest measurement errors were observed for SiC transistors mounted on a heat sink. Theoretical considerations also showed that the error would be even greater for devices cooled using liquid systems.

This issue is especially relevant for wide-bandgap transistors (SiC, GaN), which are characterized by short thermal time constants. For these devices, operating with highly efficient cooling systems, the error in thermal resistance measurement caused by TSEP oscillations can reach up to 75%. In contrast, for conventional silicon transistors, the error does not exceed 0.5% and is therefore negligible.

The presented results may be particularly useful for designers of measurement systems dedicated to thermal characterization of modern power semiconductor devices. They can also help developers of power electronic systems and cooling solutions properly design and test procedures, as well as verify the effectiveness of cooling systems.

The presented results demonstrate that the choice of sampling period can significantly affect measurement error, and at the same time indicate that the value of this error strongly depends on the material from which the tested transistor is made. To systematize the conducted investigations, our further research will involve obtaining measurement results for a larger number of sampling periods. Such results would perhaps allow for the formulation of an appropriate analytical relationship describing the effect of the sampling period on measurement errors.

## Figures and Tables

**Table 1:** Parameters describing the *Z_th_(t)* waveforms of the tested transistors.

Parameter	Si	SiC	GaN
Rth [K/W]	0.181	2.279	1.061
a_1_	0.192	0.177	0.045
τ_th1_ [ms]	629.35	25.9	100.97
a_2_	0.426	0.449	0.444
τ_th2_ [ms]	74.3	3.21	10.31
a_3_	0.243	0.275	0.382
τ_th3_ [ms]	11.89	0.75	2.77
a_4_	0.084	0.099	0.069
τ_th4_ [ms]	1.75	0.04	0.26
a_5_	0.034		0.060
τ_th5_ [ms]	0.2		0.04

**Table 2:** Absolute error *ΔT_j1_* of the tested transistors for selected values of power *P* and delay time t_d_.

Power P [W]	Time t_d_ [ms]	Si	SiC	GaN
10	0.1	0.075	3.183	1.008
10	0.3	0.124	5.281	1.690
10	1	0.212	9.763	3.021
10	3	0.354	15.064	5.253
25	0.1	0.189	7.957	2.519
25	0.3	0.310	13.202	4.225
25	1	0.529	24.408	7.553
25	3	0.886	37.659	13.132
50	0.1	0.377	15.914	5.039
50	0.3	0.620	26.403	8.450
50	1	1.058	48.816	15.107
50	3	1.772	75.318	26.264

**Table 3:** Absolute error *ΔT_j1_* of the tested transistors for selected values of power *P* and delay time t_d_ for transistors operating at natural convection.

Power P [W]	Time t_d_ [ms]	Si	SiC	GaN
1	0.1	0.016	0.356	0.179
1	0.3	0.038	0.591	0.388
1	1	0.073	1.09	0.907
1	3	0.105	1.69	1.80
2.5	0.1	0.040	0.891	0.446
2.5	0.3	0.096	1.48	0.970
2.5	1	0.182	2.73	2.27
2.5	3	0.263	4.22	4.50
5	0.1	0.080	1.78	0.893
5	0.3	0.192	2.96	1.94
5	1	0.363	5.46	4.53
5	3	0.525	8.43	9.00

**Table 4:** Absolute error *ΔT_j1_* of the tested transistors for selected values of power *P* and delay time t_d_ for transistors situated on the heat sink characterized by *R_thc-a_* = 7 K/W.

Power P [W]	Time t_d_ [ms]	Si	SiC	GaN
4	0.1	1.18	1.84	0.708
4	0.3	1.31	3.15	1.56
4	1	1.34	6.06	3.81
4	3	1.40	9.70	7.98
8	0.1	2.37	3.68	1.42
8	0.3	2.61	6.30	3.11
8	1	2.69	12.1	7.61
8	3	2.81	19.4	16.0
12	0.1	3.55	5.53	2.12
12	0.3	3.92	9.44	4.67
12	1	4.03	18.2	11.4
12	3	4.21	29.1	24.0

**Table 5:** Relative error *ΔT_j1_/(T_j_ − T_a_)* for the tested transistors under different cooling conditions and delay times *t_d_*.

Cooling Conditions	Time t_d_ [ms]	Si	SiC	GaN
Transistor without a cooling system (*R_th_* = 40 K/W)	0.1	0.019%	0.796%	0.252%
	0.3	0.031%	1.320%	0.422%
	1	0.053%	2.441%	0.755%
	3	0.089%	3.766%	1.313%
Transistor on a heat sink (*R_th_* = 7 K/W + *R_thj-c_*)	0.1	0.105%	3.430%	1.250%
	0.3	0.173%	5.691%	2.096%
	1	0.295%	10.522%	3.748%
	3	0.494%	16.234%	6.516%
Ideal cooling of the transistor case (*R_th_* = *R_thj-c_*)	0.1	4.168%	13.97%	9.498%
	0.3	6.851%	23.17%	15.93%
	1	11.69%	42.84%	28.48%
	3	19.58%	66.10%	49.51%

**Table 6:** Relative error δ_Rth_ of the tested transistors under two cooling conditions and different sampling periods.

Cooling Conditions	T_S_ [μs]	Si	SiC	GaN
Transistor without a heat sink	20	0.201%	9.84%	1.29%
	100	0.376%	10.31%	4.72%
Transistor on the heat sink	20	3.01%	9.47%	3.20%
	100	3.14%	19.06%	9.57%

## Data Availability

All data are included in the article.
